# Predictive value of frailty and nutritional status for hospital acquired pneumonia in elderly patients undergoing vascular surgeries

**DOI:** 10.3389/fmed.2025.1688157

**Published:** 2025-09-26

**Authors:** Liping Shen, Kun Wang, Feiya Wen

**Affiliations:** ^1^Department of Vascular Surgery, Zigong First People’s Hospital, Zigong, China; ^2^Department of Quality Control, Zigong First People’s Hospital, Zigong, China

**Keywords:** hospital acquired pneumonia, frailty, nutritional status, vascular surgery, elderly

## Abstract

**Background:**

Hospital acquired pneumonia (HAP) is one of the most common types of nosocomial infections, and closely related to poor prognosis. The present study intends to explore the predictive value of preoperative frailty and nutritional status for HAP in elderly patients undergoing vascular surgeries.

**Methods:**

All elderly patients who underwent vascular surgeries from June 2022 to June 2025 were retrospectively selected. Patients were divided into HAP group or non-HAP group based on the presence of HAP during the hospital stay. The frailty was assessed by using the FRAIL scale, while The nutritional status was assessed by using the Controlling Nutritional Status (CONUT) score.

**Results:**

A total of 675 elderly patients were included in the present study, of which 102 individuals were diagnosed as HAP. The proportions of smoking, general anesthesia and transfusion in HAP group were significantly higher compared with those in non-HAP group. Patients with HAP were more likely to have preoperative frailty. Significant difference in the preoperative nutritional status could also be observed between HAP group and non-HAP group. Logistic regression analysis revealed smoking, general anesthesia, frailty and moderate or severe malnutrition were independent risk factors for HAP. By using receiver operating characteristic (ROC) curve, the AUC of frailty and moderate or severe malnutrition was, respectively, 0.670 (0.606–0.734) and 0.675 (0.612–0.738). Once combined, the AUC increased up to 0.763 (0.702–0.824) with sensitivity of 0.647 and specificity of 0.838.

**Conclusion:**

HAP is a common complication among elderly patients undergoing vascular surgery. Preoperative frailty and malnutrition are independent risk factors for HAP, and have a concrete predictive efficacy. Our findings provide a basis for risk stratification and preventive measures of HAP.

## 1 Introduction

Hospital acquired pneumonia (HAP) is one of the most common types of nosocomial infections. It refers to pneumonia that occurs 48 h after patients are admitted to the hospital, which do not exist or are not in the incubation period of infection at the time of admission (1). These lesions are caused by various pathogenic microorganisms such as bacteria, fungi, and viruses within the hospital. It has been reported that HAP not only prolongs hospital stay and raises medical costs, but is also closely associated with increased mortality (2). With the acceleration of the global aging process, the number of elderly patients undergoing vascular surgeries has significantly increased. Due to the atypical clinical symptoms of respiratory system diseases in the elderly, as well as their frequent and repeated hospitalizations, fungal infections and the emergence of drug-resistant bacteria have made the diagnosis and treatment of HAP in the elderly even more challenging (3). Therefore, early and accurate identification of HAP is an urgent need for rapid recovery of elderly patients undergoing vascular surgeries.

Frailty refers to a state where multiple system functions and body’s reserve capacity gradually decline, thereby increasing the body’s susceptibility to adverse outcomes (4). It reflects the heterogeneity of health in the elderly (5). A 10-year study has shown that frailty is the most common factor leading to death (27.9%) (6). It is associated with long-term hospital stays, planned discharge, disability and other perioperative complications (7). In recent years, frailty in the elderly has gradually been incorporated into preoperative assessment, and has achieved good clinical results. A prospective cohort study involving 1,020 patients who underwent aortic valve replacement surgery found that frailty was a strong predictor of one-year mortality and disability rate (8). The pathophysiology of frailty is complex and multifactorial, and nutritional status is an important mechanism and specific target for treatment. The assessment of preoperative nutritional status has also gradually gained attention. Previous literature revealed that preoperative malnutrition significantly affected surgical outcomes, including higher rates of postoperative infections, delayed wound healing and longer hospital stays (9). Therefore, frailty and nutritional status is expected to become effective predictors of clinical prognosis.

In the present study, we retrospectively collected clinical data of elderly patients who underwent vascular surgeries in order to analyze risk factors for postoperative HAP. Furthermore, frailty and nutritional status of all included patients were evaluated. Through exploring the predictive value of preoperative frailty and nutritional status for HAP, the present study intends to identify postoperative complications in elderly patients undergoing vascular surgery at an early stage, thereby improving the clinical prognosis.

## 2 Materials and methods

### 2.1 Study design

This retrospective study was conducted in the department of vascular surgery of a single tertiary hospital in southwestern China. All elderly patients who underwent vascular surgeries from June 2022 to June 2025 were selected. This study was approved by the Ethics Committee of Zigong First People’s Hospital in accordance with the Helsinki Declaration (approval No. M2025-050). Informed consent was obtained from all included subjects.

Inclusion criteria: (a) elderly patients aged over 60 years old; (b) all patients underwent vascular surgery during their hospital stay, including open and endovascular procedures; (c) the total hospital stay was equal to or greater than 5 days, and the postoperative hospital stay was longer than 48 h. Exclusion criteria: (a) there was already a concurrent pulmonary infection at the time of admission; (b) patients who received repeated surgeries during the hospitalization period; (c) patients who were receiving nutritional therapy as an adjunct treatment upon admission; (d) patients with mental illnesses; (e) incomplete clinical data.

### 2.2 Data collection

Clinical data were extracted from the electronic medical record system or recorded manually by medical staff. Preoperative data included age, gender, body mass index (BMI), stroke, hypertension, diabetes, smoking, left ventricular ejection fraction (LVEF), albumin, total lymphocyte and total cholesterol. Intraoperative and postoperative data included general anesthesia, transfusion, operation time and postoperative bed rest time.

### 2.3 Diagnostic criteria

According to the Clinical Practice Guidelines by the Infectious Diseases Society of America and the American Thoracic Society (10), HAP was diagnosed by the clinical, imaging or microbiological evidence. Patients were divided into HAP group or non-HAP group based on the presence of postoperative HAP during the hospital stay.

The frailty was assessed by using the FRAIL scale, which was developed by the International Association of Nutrition and Aging (11). The scale consisted of five items, including fatigue, resistance, ambulation, illness, and loss of weight. Each component was a yes-or-no question, scoring one point. The total score ranged from 0 to 5, and higher scores indicated greater degree of frailty. Once the score was over 2 points, the patient was regarded as frailty.

The nutritional status was assessed by using the Controlling Nutritional Status (CONUT) score (12). As shown in Table 1, the CONUT score was calculated based on laboratory parameters, including serum albumin, total lymphocyte count and total cholesterol. The total score ranged from 0 to 12, and higher scores indicated worse nutritional status. Once the score was over 4 points, the patient was regarded as moderate or severe malnutrition.

**Table 1:** Controlling nutritional status (CONUT) score calculation.

Variables	Malnutrition			
	Normal	Mild	Moderate	Severe
Albumin				
Level (g/L)	≥ 35.0	30.0–34.9	25.0–29.9	<25.0
Score	0	2	4	6
Total lymphocyte				
Level (/mm3)	≥ 1,600	1,200–1,599	800–1,199	<800
Score	0	1	2	3
Total cholesterol				
Level (mg/dL)	≥ 180	140–179	100–139	<100
Score	0	1	2	3
Total score	0–1	2–4	5–8	9–12

### 2.4 Statistical analysis

Statistical analysis was performed by using SPSS software v25.0 (IBM corporation, United States). Kolmogorov–Smirnov test was used to evaluate the normality of measurement data. Abnormally distributed measurement data were expressed as median (Q1, Q3), and compared between groups by using Mann Whitney U test. Enumeration data were compared between groups by using Chi-square test. Taking the occurrence of HAP as the dependent variable, Logistic regression analysis was used to explore the independent risk factors. By using receiver operating characteristic (ROC) curve, predictive efficacy was compared with calculating the area under the curve (AUC). *p* < 0.05 was considered as significantly statistical different.

## 3 Results

### 3.1 Univariate analysis for HAP

A total of 675 elderly patients were included in the present study, of which 102 individuals were diagnosed as HAP, accounting for 15.11%. As shown in Table 2, the proportions of smoking, general anesthesia and transfusion in HAP group were significantly higher compared with those in non-HAP group (*p* < 0.05). Patients with HAP were more likely to have preoperative frailty (*p* < 0.05). Significant difference in the preoperative nutritional status could also be observed between HAP group and non-HAP group (*p* < 0.05). HAP group had a significant higher proportion of moderate or severe malnutrition than non-HAP group. There was no significant difference in age, male, BMI, stroke, hypertension, diabetes, LVEF, operation time and postoperative bed rest time.

**Table 2:** Comparisons of clinical characteristics between HAP group and non-HAP group.

Variables	HAP group (*n* = 102)	Non-HAP group (*n* = 573)	*p* value
Age (y)	72.50 (66.00–77.25)	69.00 (65.00–77.00)	0.281
Male	59	341	0.744
BMI (kg/m^2^)	23.37 (20.79–26.67)	23.26 (20.80–25.93)	0.484
Stroke	7	23	0.195
Hypertension	35	189	0.415
Diabetes	42	244	0.662
Smoking	28	101	0.028*
General anesthesia	30	98	0.006*
Transfusion	17	44	0.008*
LVEF			0.076
≤ 50%	13	42	
> 50%	89	531	
Operation time			0.266
≤ 2 h	70	359	
> 2 h	32	214	
Postoperative bed rest time			0.107
≤ 2d	87	520	
> 2d	15	53	
Frailty	44	52	<0.001*
Malnutrition			<0.001*
Normal, mild	54	504	
Moderate, severe	48	69	

HAP, Hospital acquired pneumonia; BMI, Body mass index; LVEF, Left ventricular ejection fraction. *Indicates difference is statistically significant.

### 3.2 Multivariate analysis for HAP

Six factors with *p* value less than 0.1 were taken as independent variables. By using Logistic regression analysis, smoking, general anesthesia, frailty and moderate or severe malnutrition were found to be independent risk factors for HAP (*p* < 0.05, Table 3).

**Table 3:** Logistic regression analysis of independent risk factors for HAP.

Variables	B	SE	Wals	*p*	OR
Smoking	0.926	0.290	10.175	0.001*	2.525
General anesthesia	0.894	0.293	9.325	0.002*	2.444
Transfusion	0.700	0.368	3.607	0.058	2.013
LVEF ≤ 50%	0.592	0.400	2.193	0.139	1.808
Frailty	1.844	0.279	43.575	0.000*	6.320
Moderate or severe malnutrition	1.666	0.269	38.403	0.000*	5.289

HAP, Hospital acquired pneumonia; LVEF, Left ventricular ejection fraction. *Indicates difference is statistically significant.

### 3.3 Predictive value of frailty and nutritional status for HAP

**Figure 1:**
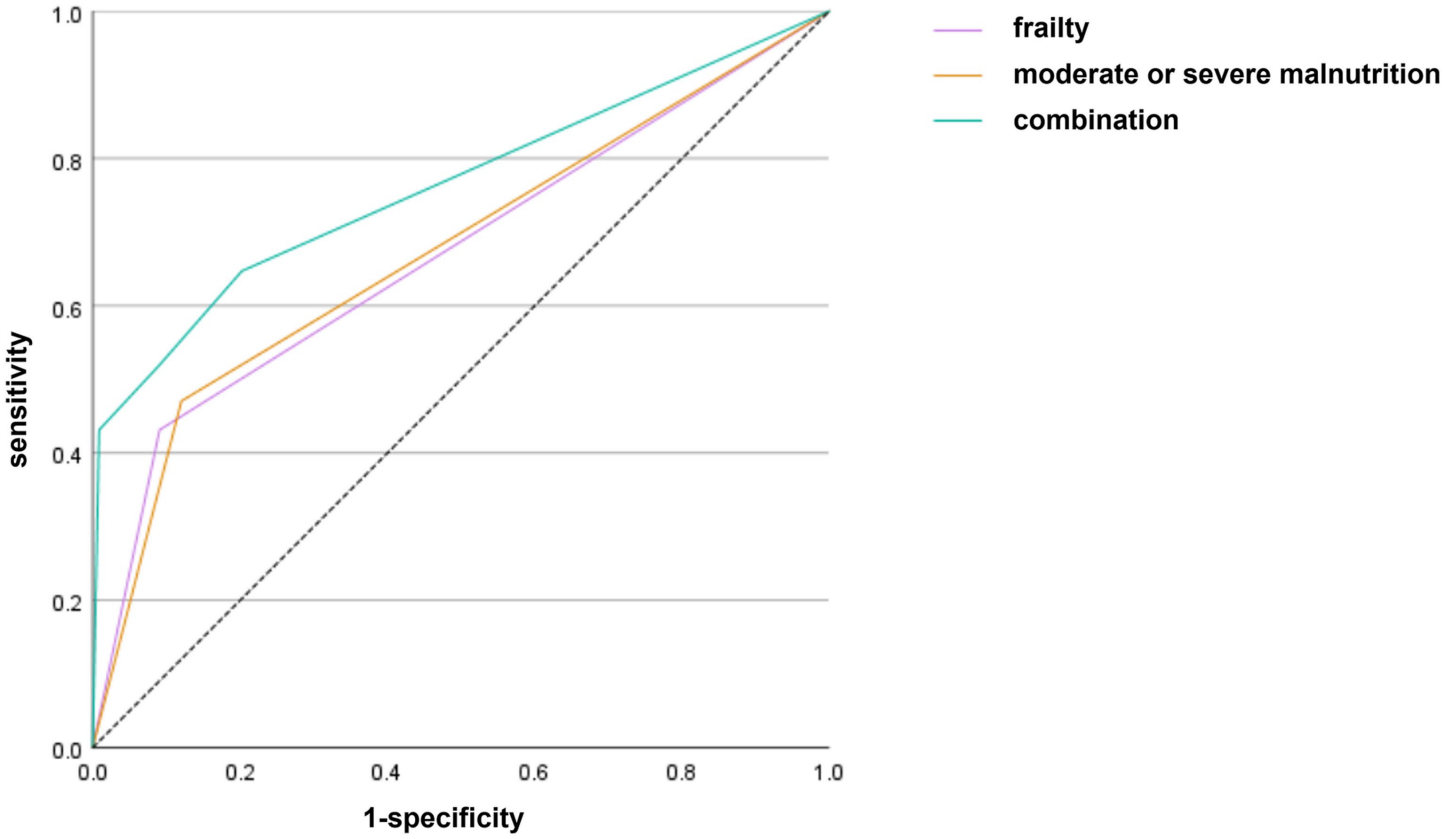
ROC curve of frailty and malnutrition in predicting HAP.

Table 4 and Figure 1 showed the ROC curve results of frailty and malnutrition in predicting HAP. The AUC of frailty was 0.670 (0.606–0.734) with sensitivity of 0.431 and specificity of 0.909 (*p* < 0.05), whereas the AUC of moderate or severe malnutrition was 0.675 (0.612–0.738) with sensitivity of 0.471 and specificity of 0.880 (*p* < 0.05). Once frailty was combined with moderate or severe malnutrition, the AUC increased up to 0.763 (0.702–0.824) with sensitivity of 0.647 and specificity of 0.838 (*p* < 0.05).

**Table 4:** Predictive efficacy of frailty and nutritional status for HAP by ROC curve.

Variables	AUC (95% CI)	Sensitivity	Specificity	*p* value
Frailty	0.670 (0.606–0.734)	0.431	0.909	0.000*
Moderate or severe malnutrition	0.675 (0.612–0.738)	0.471	0.880	0.000*
Frailty + Moderate or severe malnutrition	0.763 (0.702–0.824)	0.647	0.838	0.000*

ROC, Receiver operating characteristic curve; HAP, Hospital acquired pneumonia; AUC, Area under ROC curve; CI, Confidential interval. *Indicates difference is statistically significant.

## 4 Discussion

Hospital-acquired infections are responsible for an estimated 1.4 million deaths worldwide each year based on reports from the World Health Organization (WHO), of which HAP is one of the most common types (13). Our present study found that the prevalence of HAP in elderly patients undergoing vascular surgeries was up to 15.11%. Elderly patients generally have degenerated immune function, reduced strength of respiratory muscles, and weakened cough reflex, resulting in decreased ability to expel phlegm (14). At the same time, they often have multiple underlying diseases, further reducing their ability to withstand infections. These factors make elderly patients themselves a high-risk group for HAP. Vascular surgery is mostly a type of operation with significant trauma. After the surgery, patients often need to stay in bed for a long time due to pain, limb immobilization or vascular complications, thus leading to insufficient lung ventilation and accumulation of sputum (15). Moreover, vascular diseases are often accompanied by limb ischemia, which may indirectly cause pulmonary infections through hematogenous dissemination or systemic inflammatory responses (16). The above factors may constitute the pathophysiological basis for the occurrence of HAP in elderly patients undergoing vascular surgery.

Frailty is a psychobiological syndrome related to age, manifested as the decline of physical, cognitive, social, and psychological function. Nowadays, frailty has become a global health burden. A meta-analysis covering 62 countries shows that the prevalence of frailty among people aged over 50 is 24%, and the prevalence of pre-frailty is as high as 49% (17). Frailty is considered to be closely related to poor clinical outcomes. Therefore, clinical guidelines recommend that elderly patients should undergo a frailty assessment before surgery (18). Pooled analysis from 16 studies in patients with vascular diseases found significant associations between 30-day mortality and frailty (17). Frailty is also a strong predictor of long term outcomes in vascular surgery, such as 5 year mortality (19). In the present study, we found that preoperative frailty was an independent factor for HAP. The immune and respiratory dysfunction caused by frailty may be the main mechanism leading to HAP. Besides, The swallowing reflex latency of frail elderly people is prolonged, and the contraction force of the pharyngeal muscles is weakened, resulting in incomplete closure of the epiglottis during swallowing (20). As a result, gastric contents or oral secretions are prone to reflux into the airway, leading to the occurrence of HAP. At present, there are various tools available for assessing frailty in the elderly. Our present study used the FRAIL scale as the assessment instrument. The FRAIL scale does not require any physical examination, making it convenient for medical staff to make evaluation. Compared with other tools, it is more convenient and efficient, and can accurately assess the patient’s health condition. The Chinese version of FRAIL scale has been proved to present acceptable validity and reliability (21). However, it is still important to make concern that the FRAIL scale mainly relies on the subjective description of patients’ feelings, and there may be certain inaccuracies.

In this study, the CONUT score was used to systematically assess the preoperative nutritional status of elderly patients undergoing vascular surgery. It was found that moderate to severe malnutrition was an independent risk factor for HAP, and exhibited significant predictive efficacy. This result not only confirms the crucial impact of nutritional status on postoperative infectious complications, but also provides important evidence-based support for perioperative risk management of elderly patients. Compared to single nutritional indicators, the CONUT score, as a comprehensive nutritional assessment tool including three indicators, demonstrates greater efficacy in predicting postoperative clinical outcomes (22, 23). Albumin is not only one of the indicators for evaluating the nutritional status of the human body, but also an important factor in regulating acute inflammatory responses. Low lymphocyte counts directly indicate immunodeficiency and have a strong correlation with the pathology of HAP. Cholesterol reflects energy reserves and is more in line with the metabolic characteristics of the elderly. The three parameters of CONUT score precisely cover the core pathways of HAP - albumin (mucosal repair / osmotic pressure), lymphocytes (immune activity), and cholesterol (energy / hormone synthesis), thus having a high degree of predictive accuracy (24). Furthermore, the CONUT score provides a practical risk stratification tool for clinical use through clear numerical classification. This quantitative characteristic makes it easier to integrate into the preoperative assessment process for elderly patients, assisting in the formulation of individualized treatment plans. Although the AUC values for frailty and malnutrition were 0.670 and 0.675, respectively, indicating moderate predictive efficacy, their combination yielded an AUC of 0.763, which is clinically meaningful. Single indicator screening may lead to missed diagnoses due to its limitations. Combined testing with frailty and malnutrition, by covering multiple dimensions of pathological mechanisms, can identify high-risk individuals more precisely and provide a time window for intervention.

The key to reducing the adverse clinical effects of HAP lies in establishing a refined infection control strategy. At present, various effective preventive measures for HAP have been reported. Hand hygiene was considered a very important strategy to prevent HAP (25). Regularly conducting oral care is also beneficial for reducing the number of bacteria in the mouths, thus reducing the occurrence of HAP (26). Some other care bundles, such as reverse Trendelenburg position and dysphagia screening (27), might be potential preventive approaches, but further verification is still needed. The results of this study show that preoperative frailty combined with malnutrition can effectively predict HAP in elderly patients undergoing vascular surgery, which provides an important basis for clinical formulation of targeted intervention strategies. Malnutrition is the primary cause of low immune function in elderly patients, and the frail state further aggravates metabolic disorders and the decline in tissue repair capacity. Preoperative nutritional support should be regarded as the first step of intervention. For patients with moderate to severe malnutrition, nutritional status can be improved before surgery through oral nutritional supplements or enteral tube feeding. Additionally, individualized plans should be formulated based on the patient’s swallowing function and digestive absorption capacity to avoid adverse events such as abdominal distension and aspiration caused by excessive feeding. Furthermore, preoperative targeted intervention for frailty could significantly improve the physical condition and stress tolerance of patients. Exercise rehabilitation training is the core method. For patients with frailty, low-intensity interventions such as passive joint movement and bedridden limb positioning can be adopted to prevent muscle atrophy. At the same time, attention should be paid to the psychological state of elderly patients. Through family support and cognitive behavioral intervention, anxiety and depression can be alleviated to avoid immune suppression caused by psychological stress. In clinical practice, personalized regimens should be formulated based on individual patient conditions. Continuous monitoring of relevant risk factors should be carried out, and intervention strategies should be constantly optimized.

There still exist some limitations in the present study. First, the single-center retrospective design inevitably introduces selection bias and measurement bias, and the sample representativeness may be limited. Although some confounding factors were corrected through multivariate regression, unrecorded confounding factors (such as specific surgical procedures, antibiotic usage, etc.) may still affect the association effect. The predictive efficacy of the ROC curve is generally poor, and further prospective studies are needed for optimization before clinical promotion.

## 5 Conclusion

HAP is a common complication among elderly patients undergoing vascular surgery. Our study demonstrates that preoperative frailty and malnutrition are independent risk factors for HAP, and their combination has a concrete predictive efficacy. This result not only provides a basis for risk stratification of HAP in elderly patients, but also reveals the key mechanism of postoperative infection susceptibility, holding significant clinical and theoretical significance. Future studies should focus on the possibility of comprehensive intervention measures in reducing the risk of HAP, such as preoperative nutritional optimization, improvement of frailty status, and multidisciplinary collaborative management.

## Data Availability

The raw data supporting the conclusions of this article will be made available by the authors, without undue reservation.
